# Wiener filter improves diagnostic accuracy of CAD SPECT images—comparison to angiography and CT angiography

**DOI:** 10.1097/MD.0000000000014207

**Published:** 2019-01-25

**Authors:** Michael A. Masoomi, Iman Al-Shammeri, Khaled Kalafallah, Hany M.A. Elrahman, Osama Ragab, Ebba Ahmed, Jehan Al-Shammeri, Sharif Arafat

**Affiliations:** a Department of Nuclear Medicine and Molecular Imaging, Adan HospitalHadiya, KW; b Department of Nuclear Medicine, Kuwait Cancer Control Centre, Sabah Medical District, Shuwaikh; c Department of Nuclear Medicine, Faculty of Medicine, Heath Science Centre, Kuwait University; d Department of Cardiology, Dabbous Cardiac Centre, Adan Hospital, Hadiya, Kuwait.

**Keywords:** angiography, computerized tomography, quantitative and qualitative imaging, reconstruction filters, single photon emission computerized tomography

## Abstract

Many discrepancy in selection of proper filter and its parameters for individual cases exists. The authors investigate the impact of the most common filters on patient NM images with coronary artery disease (CAD), and compare the results with the computerized tomography (CT)-Angio and angiography for accuracy.

The investigation initiated by performing various single photon emission computerized tomography (SPECT)/CT scan of the national electrical manufacturers association chest phantoms having hot and cold inserts. Data acquired on GE 670 PRO SPECT/CT; 360Ø, 64 frames, 60 seconds, low energy high resolution (LEHR) 128, low energy general purpose (LEGP) with CT attenuation (120 kV and 170 mA). The images reconstructed with filtered back projection and ITERATIVE ordered-subset expectation maximization utilizing filters; Hann, Butterworth, Metz, Hamming, and Wiener. The Image contrast was calculated to assess absolute nearness of the inserts. Based on the preliminary results, then scans of 92 patients with CAD; 64 males and 28 females, age 41 to 77 years old, who had been reported earlier reprocessed with the nominated filter and were reported by 2 NM expert. The results compared to the earlier reports and to the CT–Angio and angiography.

The optimization suggested 3 filters; Wiener (Wi), Metz and Butterworth (But) provide the highest contrast (99– 66.4%) and (81– 32%) for the cold and hot inserts respectively, with the (Wi) filter to be the better option. The reprocessed patients scan with the (Wi) presented an elevated diagnostic accuracy, correlated well with the CT-Angio and angiography results (*P* < .001 and *r* = 0.79 for [Wi] and *P* = .004 and *r* = 0.39 for [But]). The percentage of the false negative for moderate to severe CAD cases reported using Wi filter reduced from 27% to 7% and similarly for mild CAD cases from 7% to 1%.

It appears the Wiener filter could produce results with the highest contrast for phantom imaging of various cold and hot spheres and for the patient data which is more consistent with angiography results, with much-elevated accuracy in intermediate cases (*r* = 0.79 for Wiener and *r* = 0.39 for Butterworth vs angiography). However, the optimum parameters obtained for the filters have no relation with the resolution of the imaging system, but the details of the objects could be improved.

## 1 Introduction

Myocardial perfusion imaging (MPI) using single photon emission computerized tomography (SPECT) ^99m^Tc-sestamibi is an accurate noninvasive means of detecting coronary artery disease (CAD) and assessing the severity of perfusion abnormalities in patients with coronary stenosis. Low-pass filters smoothen the image, obscure the significance of small lesions, and reduce the sensitivity of the technique.^[1,2]^ Restoration filters, on the other hand, enhance the image contrast, exaggerate artifacts at certain frequencies, and reduce the specificity of the technique.^[3,4]^ Many discrepancy in selection of proper filter and its parameters for individual cases exists. Patients with normal SPECT MPI have a very low cardiac event rate estimated at <1% per year.^[5–8]^ Although rare, left main (LM) CAD, and balanced multivessel disease (MVD) can result in a falsely normal MPI study despite the high associated cardiovascular risk. Only a fraction of patients with CAD involving the LM artery or MVD have perfusion abnormalities in all the coronary artery territories on MPI.^[9–11]^

Most SPECT filter functions allow the user to control the degree of high-frequency suppression by choosing a cutoff frequency, or similar filter parameter, which determines where the filter rolls off to 0 gain. There should exist an optimum cutoff frequency for a particular filter function which compromises the trade-off between noise suppression and spatial resolution degradation. SPECT filters can greatly affect the quality of clinical images by their degree of smoothing. Determining the best filter and the proper degree of smoothing can help to ensure the most accurate diagnosis.

Noise reduction is an important part of data processing in SPECT imaging.^[12–14]^ The extent and distribution of noise in tomographic images are very much dependent upon the method of reconstruction being used.^[15,16]^ Despite the fact that there are new techniques of image reconstruction, the most widely used method is still filtered back projection (FBP) method due to simplicity and speed.^[17,18]^ The main drawback of FBP is the noise.^[19–21]^ Unfortunately, there have been significant discrepancies in the selection of proper filter and adjustment of the filter parameters to individual cases.^[22–25]^ Different authors and different manufactures suggested different filters.^[26,27]^ Though much work has been done, there are still considerable inconsistencies.

Heart diseases are the primary cause of death in Kuwait, representing more than 40% of the total deaths annually. The rate of heart diseases among women in Kuwait does not exceed 15%, but there is an increase in the causes of heart disease in Kuwait, where a recent local study has shown that overweight and obesity has increased to 60% according to the KW-MOH statistic.

Computed tomography coronary angiography (CTCA) is a technique proved to provide high sensitivity and negative predictive value for the identification of anatomically significant CAD when compared with invasive X-ray coronary angiography.^[28,29]^ While the CTCA limitation of an ionizing radiation dose delivered to patients is substantially overcome by recent technical innovations, a relevant limitation remains the only anatomical assessment of coronary stenosis in the absence of evaluation of their functional hemodynamic significance. This limitation is highly important for those stenosis graded as intermediate at the anatomical assessment and it often overestimates the clinical relevance of stenosis. In particular, even if most CTCA-detected coronary stenosis is confirmed at CCA, less than half of those studied with FFR cause myocardial ischemia.^[30,31]^ Thus, at least a percentage of stenosis could be overtreated: the revascularization of such lesions would provide no clinical benefit in terms of improvement of blood flow but exposes the patient to the risks of this procedure.^[32]^

In this study, we instigated to assess processing methods at the locality comparing 5 widely used filters; Hanning, Butterworth, Metz, Hamming, and Wiener initially together with FBP and Iterative (with ordered-subset expectation maximization [OSEM]) reconstruction methods for selecting the optimized filter for reconstruction of myocardial ^99m^Tc-sestamibi SPECT studies of patients with CAD. The results then compared with the computerized tomography (CT)-Angio and the invasive angiography of the patients for further evaluation and to improve quantitative and qualitative accuracy of the images though, the authors are addressing the quantitative impact of the proposed filters in more detail in a separate study that is currently ongoing and will be published separately in very near future.

## 2 Materials and methods

### 2.1 Phantom

**Figure 1:**
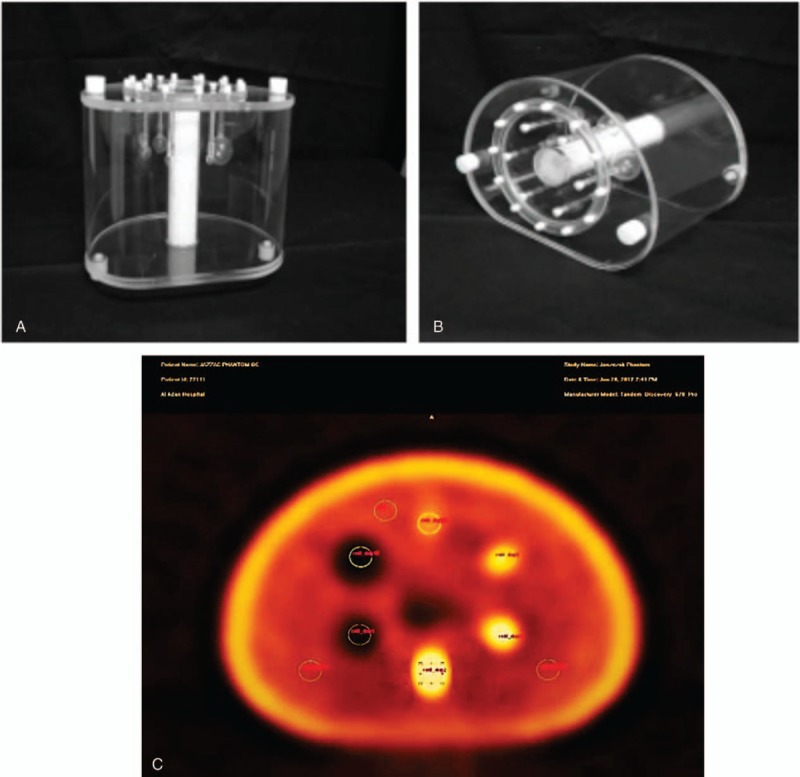
The NEMA phantom (cold and hot spheres) and the transaxial image. NEMA = national electrical manufacturers association.

The investigation initiated by performing the multiple SPECT/CT scans of a national electrical manufacturers association chest phantom (Fig. 1) having hot and cold inserts (10, 13, 17, 22, 28, and 37 mm). Data acquired on GE 670 PRO SPECT/CT; 360Ø, 64 frames, 60 seconds, 128, LEHR with CT attenuation (120 kV and 170 mA). Due to the variation of imaging system structure and to acquire statically reliable and acceptable images, the optimized activity was calculated as follows (Equation 1). 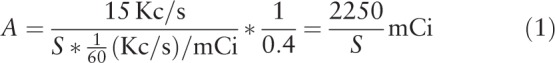


The images were reconstructed on GE Xeleris workstations using the available and mostly utilized reconstruction algorithms locally; FBP and ITERATIVE OSEM (2 iterations and 8 subsets) and the selected common filters; Hanning, Butterworth, Metz, Hamming, and Wiener (Equation 2). The Image contrast [C = (*R*0–*R*1)−/*R*0 ∗ 100] where *R*0 and *R*1 are accumulated counts in a defined ROI over the mid-slice of inserts as well as on the background, was calculated for each set of the reconstructed image of the phantom utilizing the stated filters, to assess absolute nearness of the inserts quantitatively and qualitatively. 
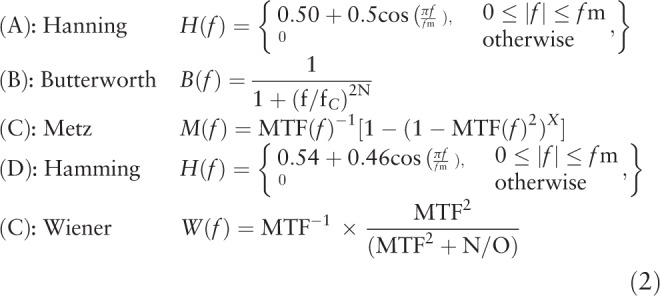


### 2.2 Patients

All patients were referred for SPECT/CT imaging, from a nearby specialized chest hospital and the Dabbous cardiac center which is a large local cardiovascular center. All patients as part of the routine clinical study had signed the consent form. The ethical approval was not necessary, though reprocessing of patient data was approved by the department as part of optimization.

The referral patients had their height and weight measured, and their body mass indexes were calculated. All patients underwent initial ^99m^Tc-Myoview stress-first SPECT. Immediately after the acquisition of stress SPECT scan, a cardiologist and a nuclear physician together assessed the need for additional rest SPECT imaging. In case of abnormal stress perfusion, additional rest SPECT is performed. Stress testing was routinely performed with pharmacological stress using adenosine (140 μg min^−1^ kg^−1^ for 6 minutes) in all patients unless there was a contraindication for pharmacological stress. Patients were instructed to refrain from caffeine-containing beverages for at least 24 hours before the test. In case of a contraindication for adenosine, patients underwent dobutamine (starting dose of 10 μg kg^−1^ min^−1^, increased at 3-minute intervals to a maximum of 50 μg kg^−1^ min^−1^), or treadmill testing. A weight-adjusted dose of ^99m^Tc-Myoview (standard, 740 MBq; 1000 MBq for patients >100 kg) is administered after 3 minutes (adenosine or when the target heart rate of >85% of predicted maximal was reached [dobutamine, treadmill test]). Patients scheduled for rest imaging received a dose of ^99m^Tc-Myoview (standard, 740 MBq; but 1000 MBq for patients >100 kg). Both stress and rest SPECT images were acquired 45 to 60 minutes after tracer injection with time 24 hours time delay between the stress and rest studies.

The cardiac images were reconstructed and displayed on the short, vertical, and horizontal long axis.

Scans of 92 patients (66 males and 26 females, age 41–77 years old) selected from 200 scanned patients with known or suspected CAD who meet the criteria (have had CT–Angio and Angiography) and had been reconstructed as per local setting and had been reported earlier, reprocessed with the nominated filter. Two expert nuclear medicine physicians evaluated the resulting images on the computer screen independently and were blinded from the earlier reports. All reconstructed cardiac tomography images were reevaluated with respect to the presence of a defect and 4-point scale system (1 = normal perfusion, 2 = mildly reduced uptake, 3 = moderately reduced uptake, and 4 = severe reduced uptake) for semi-quantitative assessment. Reports of the reassessed images, then compared to the CT-Angio and angiography results of the patients. All patients had ^99m^Tc-Myoview scan as per defined protocol and before the CT–Angio or Angiography, which performed in the radiology department and the Dabbous cardiac center.

### 2.3 Statistical analysis

Spearman Rho a nonparametric test used to measure the strength of association between 2 variables. The Pearson correlation coefficient was used to determine linear relation between the numerical values for the presence or absence of CAD in comparison to CT–Angio and Angiography. The data were expressed as 4-point scale system for 2 selected filters (Butterworth and Wiener). In addition, we correlated the presence or the absence of CAD against CT-Angio and angiography finding with Correlation Coefficient and *P*-values accordingly. Furthermore, true positive, true negative, false positive, false negative, sensitivity and specificity, positive likelihood ratio, and negative likelihood ration were tabulated for application of Wiener and Butterworth filters that were utilized in reprocessing of clinical patient data (Table 1). The percentage of cases reported normal or equivocal, before and after reconstruction using the optimized filters, were also reported and compared.

**Table 1:**
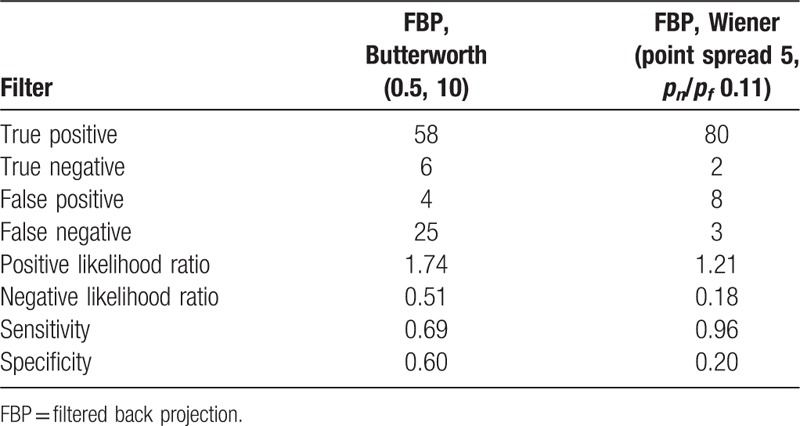
Statistical values for 2 separate filters; Butterworth and Wiener, that were used for reprocessing of patients clinical data.

## 3 Results

**Figure 2:**
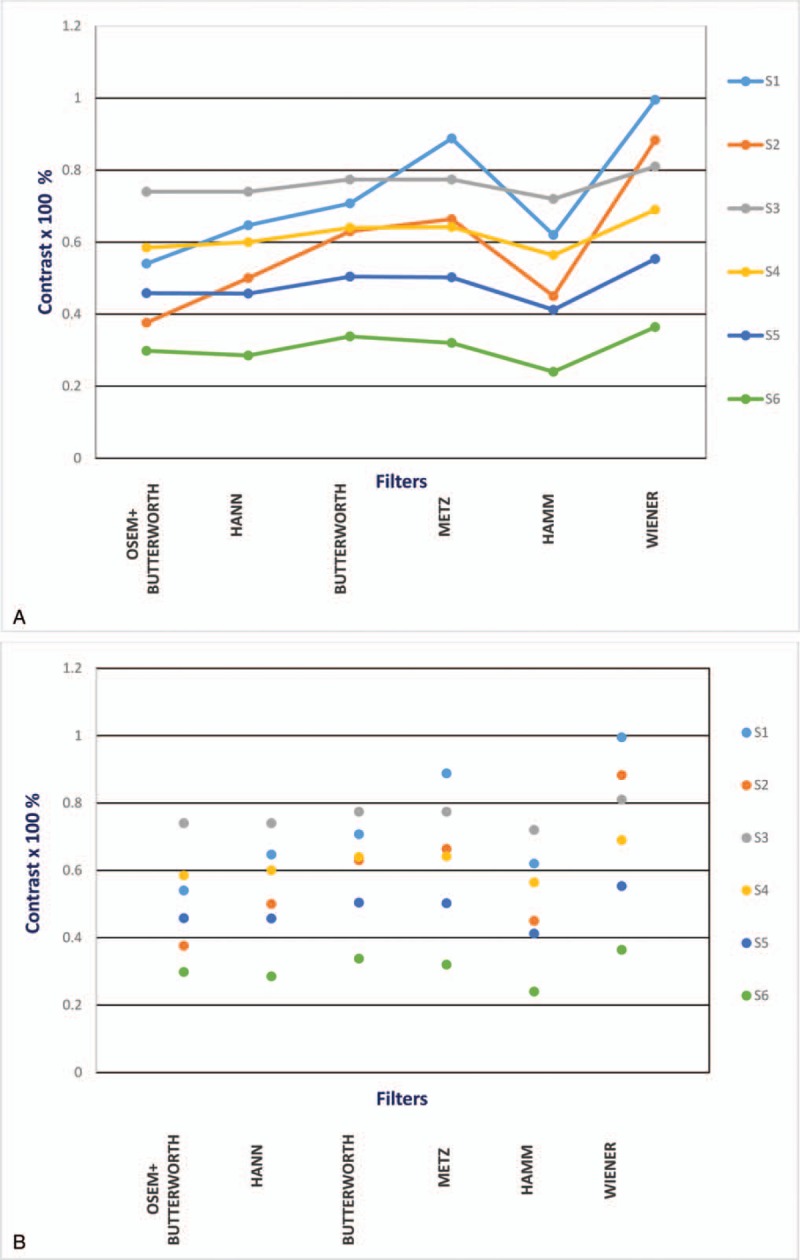
(A): Comparison of contrast for different reconstructing filters of a NEMA chest phantom: inclusive of 6 variable spheres (S1 and S2 are cold spheres) and (S3, S4, S5, and S6 are hot spheres), (B): Scattering plot for comparison of contrast for different reconstructing filters of the same NEMA phantom and the embedded spheres. NEMA = national electrical manufacturers association.

Initially, images of the body phantom utilizing the stated filters (Equation 1) were reconstructed with FBP and OSEM methods. The related parameters for each individual filter were modified to reach the optimum visual image quality with reference to the body phantom (Butterworth: cutoff frequency = 0.5 and order = 10; Hanning: cutoff frequency = 1; Metz: PSF = 5 and order = 3; Hamming: cutoff frequency = 1 Wiener; PSF = 5 and noise to signal = 0.11 ratio). The contrast for each set of reconstructed images was calculated to complement the visual assessment quantitatively (Table 2). The calculated contrast suggested 3 filters; Wiener, Metz, and Butterworth provide highest contrast values (99.5%, 88.8%, and 70.7%) for the S1 cold sphere (38 mm) and (88.3%, 66.4%, and 63%) for the S2 cold sphere (27 mm). However, for the hot spheres inserts (10, 13, 17, 22 mm), Wiener and Butterworth filters appear to provide better contrast values than the rest of reconstructed filters, with the Wiener filter is the preferred choice (Fig. 2A and B). We used the outcome of the body phantom study to repeat processing the 92 cardiac patients scan that has already been reconstructed with the Butterworth filters for comparison and ultimately to achieve better accuracy in the diagnosis of patients with CAD.

**Table 2:**
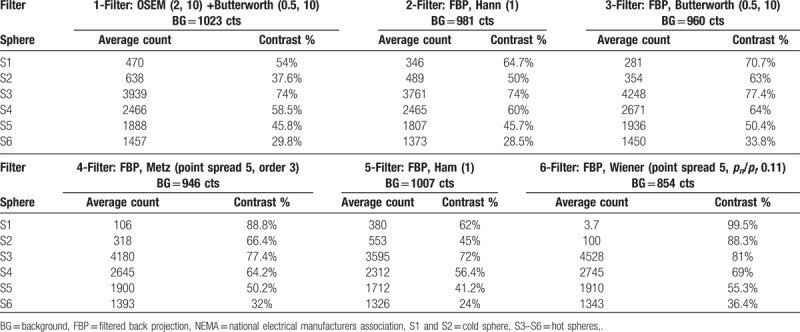
Calculated contrast values for 6 spheres (hot and cold) – NEMA chest phantom.

**Figure 3:**
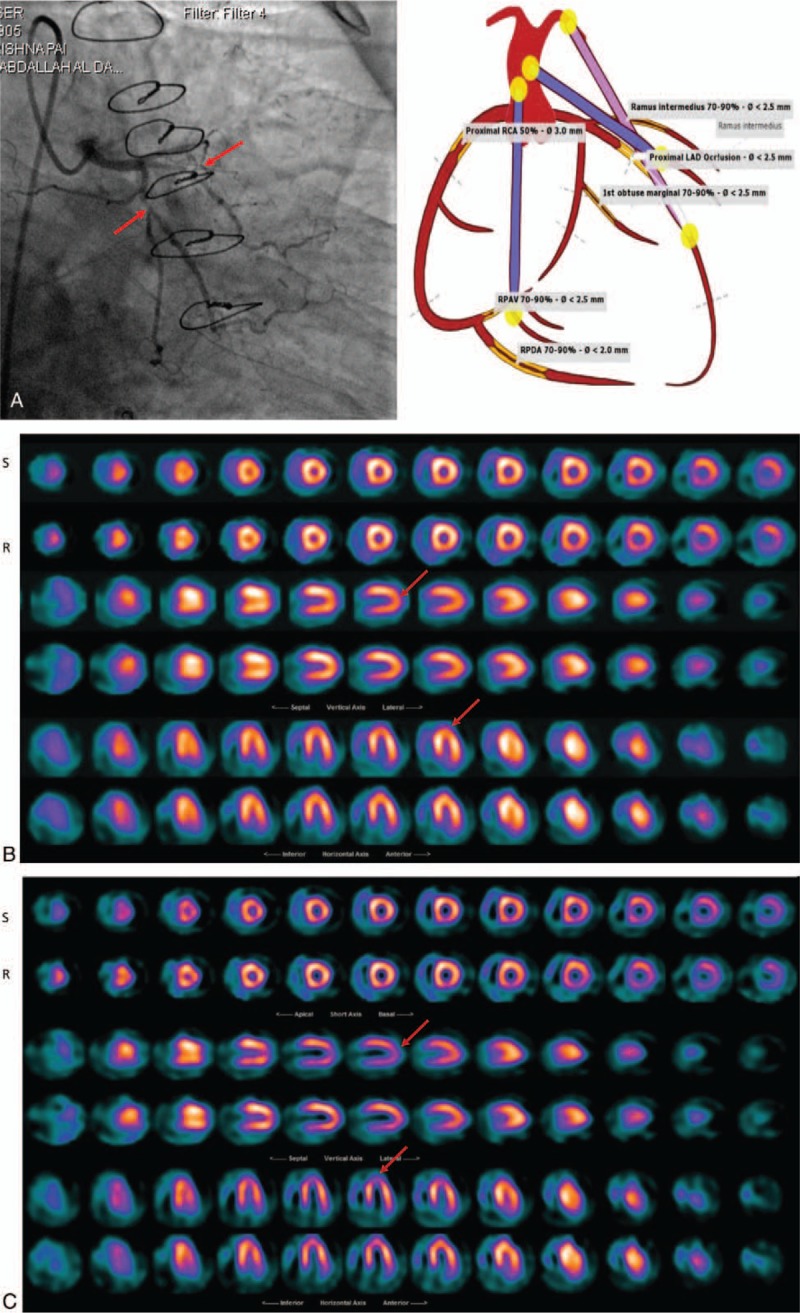
A 53 years old male patient with the history of CAD was refereed to NM for evaluation of myocardial perfusion post CABG. (A): Angiography result reported triple vessels disease, (B): Butterworth reconstructed SPECT results reported mild degree of ischemia in apex, (C): Wiener reconstructed SPECT results suggested severe diffuse uptake in anterior and anterolateral walls with moderate diffuse uptake in apex that was more consistent with angiography. CABG = coronary artery bypass grafting, CAD = coronary artery disease, SPECT = single photon emission computerized tomography.

**Figure 4:**
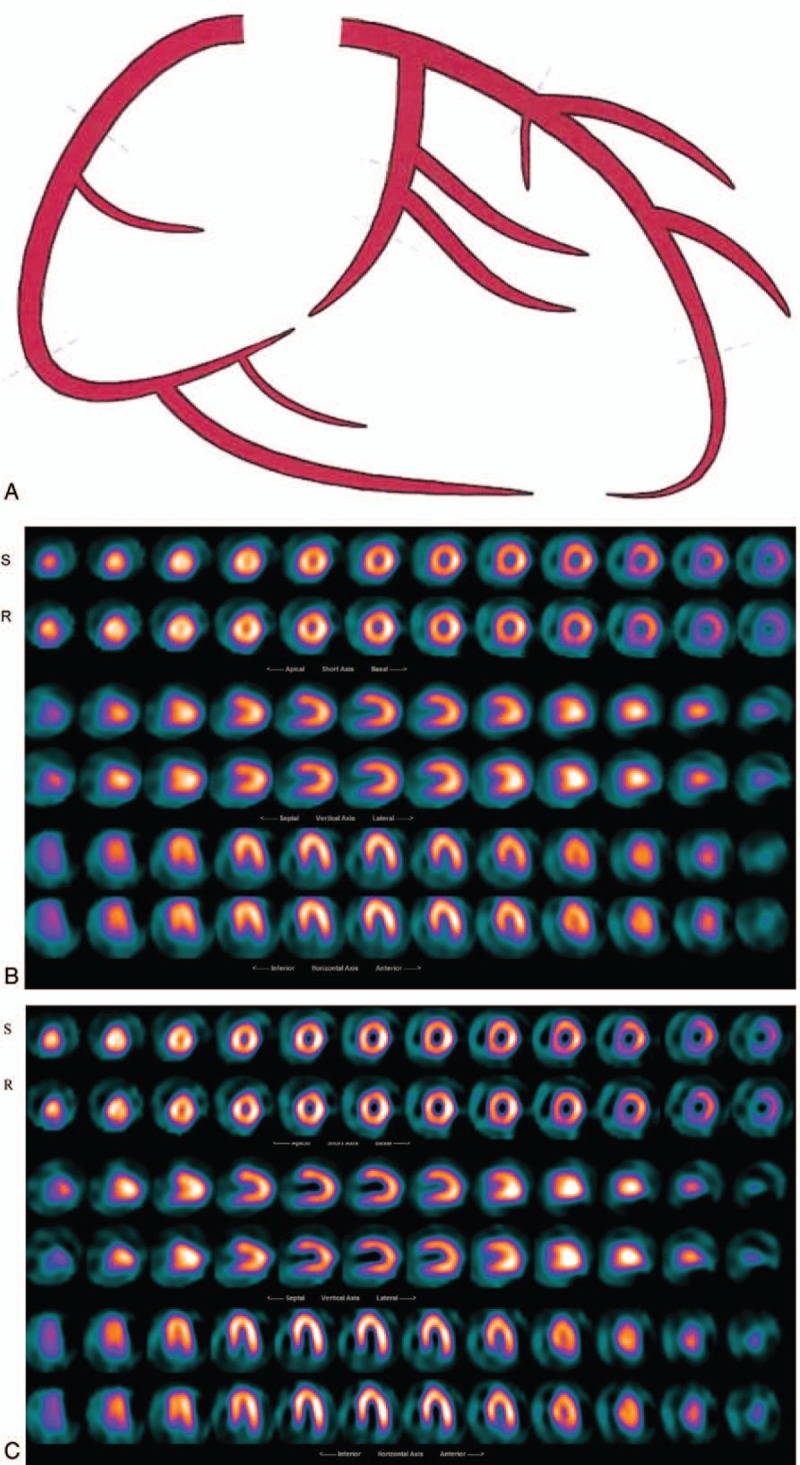
A 41 years old male patient with atypical chest pain and shortness of breath was refereed to NM for assessment of possible CAD. (A): CT-angiography result reported Normal (LMCA, LAD, Diag, Circ, and RCA are normal), (B): Butterworth reconstructed SPECT results reported normal, (C): Wiener reconstructed SPECT results suggested normal with very mild diffuse uptake in basal anterolateral wall and inferior wall (likely due to diaphragmatic attenuation). Both reports were consistent with the CT-angiography. CAD = coronary artery disease, Circ = circumflex, CT = computerized tomography, Diag = diagonal, LAD = left anterior descending artery, LMCA = left main coronary artery, RCA = right coronary artery, SPECT = single photon emission computerized tomography.

**Figure 5:**
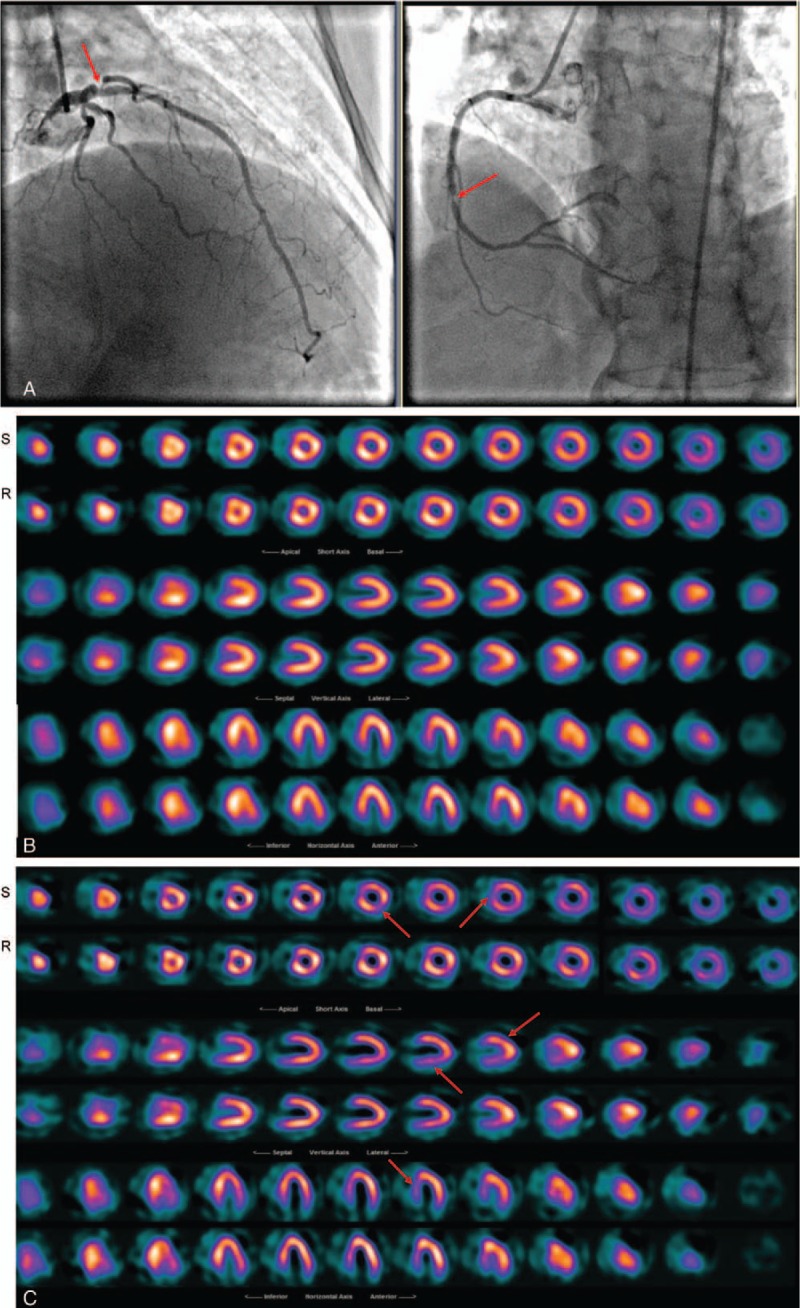
A 62 years old female patient with atypical chest pain and history of multiple PCI was referred to NM for evaluation of myocardial perfusion. (A): Angiography result reported triple vessels disease, (B): Butterworth reconstructed SPECT results reported negative myocardial ischemia, (C): Wiener reconstructed SPECT results suggested diffuse uptake in inferior wall, apical and mid anterolateral walls + cardiomyopathy in septal wall that was more consistent with the angiography. PCI = percutaneous coronary intervention, SPECT = single photon emission computerized tomography.

All 92 patients had a CT–Angio and invasive Angiography, which were set as the reference tests for comparison of the previous and the current reconstructed images using Butterworth and Wiener respectively. Scan of cardiac patients was carried out over a period of 15 months and the same imaging protocol was utilized over the period. Those patients who underwent different imaging procedure were omitted from this study to achieve constancy approach. The reprocessed patients scan with the Wiener filter presented an elevated improvement in diagnostic accuracy, correlated well with the CT-Angio and angiography results (*r* = 0.79 for Pearson and *r* = 0.78 for Spearman) and were statistically significant (*P* < .001) in comparison to the Butterworth (*r* = 0.4 for Pearson and *r* = 0.39 for Spearman with *P* = .005). The earlier report using FBP and the Butterworth suggested 25% of patients who had moderate to severe CAD and 6% who had mild CAD as normal. We reconstructed the same patient data with the Wiener filter and then reported by the same NM experts. The percentage of false negative reporting reduced to 7% for moderate to severe and 1% for the mild CAD cases in comparison to the angiography and CT-Angio results. The 4% of patients who had either angiography or CT-Angio and reported normal, also reported normal where their NM scans were reconstructed with Butterworth and Weiner (Figs. 3–5). In addition, we applied Spearman Rho a nonparametric test to measure the strength of association between 2 variables. The value of *R* was 0.78 for the images processed with Wiener filter and, similarly as the Pearson test, it was well correlated with Angio-CT and angiography and *P*-value was less than <.001 and the association between the 2 variables was considered statistically significant. Similarly, the value of *R* for the images processed with Butterworth filter was 0.39 and the *P*-value was .004. Furthermore, true positive, true negative, false positive, false negative, sensitivity, specificity, positive likelihood ratio, and negative likelihood ratio were tabulated for the Wiener and Butterworth filters which applied in the processing of clinical patient data (Table 1). Sensitivity of the Wiener filter (0.96) is suggesting a high probability that Wiener filter will correctly diagnose patient CAD in comparison to the Butterworth filter with the sensitivity of 0.69.

The quantitation aspect of filtering and its impact on the present study is subject to the on-going separate in-depth investigation. However, at this stage we are using Cedar Sinai QPS for quantitative scoring; summed stress ischemia scoring-, summed rest ischemia scoring, and summed difference ischemia scoring. The initial results are suggesting improvement in quantification using a Wiener filter with good correlation versus CAD diagnosis using the same filter (*r* = 0.7). Comparatively, the Butterworth filter is suggesting poorer correlation (*r* = 0.37), though the current outcome is based on a limited number of cases at this stage.

## 4 Discussion

The current practice for cardiac imaging in our very busy local hospital was based on FBP and (But) filter for processing the SPECT/CT images. The OSEM and (But) reconstruction filter mostly used for processing other imaging cases, though occasionally used for cardiac image processing.

The blurring caused by gamma camera is due to the fact that, it is unable to register the high-frequency components of the data. As a result, the details of the objects are not actually recorded and the images become blurred. Logically, it is not possible to compensate this type of blurring using deconvolution or any other techniques. The frequency components of the data are independent. However, the other factor that contributes to blurring is due to back-projection, which is different from the above and it is not within the remit of this project.

The selected Wiener filter has characteristics of both the smoothing and blurring compensation. It reduces the high-frequency components of the data (that are supposed to be the noise) and at the same time, some selected medial frequencies are magnified. The Wiener filter divides the frequency components of the data by MTF of the system. MTF is the normalized Fourier transform of the point-spread function (PSF). This action should compensate the blurring effect represented by MTF. Blurring caused by back-projection affects the data at a lower frequency compared to blurring caused by a gamma camera, simply because it is an excess blurring.

It should not also be forgotten that the frequency function of a low-pass filter should always match the noise distribution in the data. Wiener filter is designed to minimize the mean square-error between filtered image and true image. Noises usually have constant amplitudes at all frequencies (white frequency spectrum) but data has normally higher amplitudes at low frequencies and lower amplitudes at higher frequencies.

During the process of optimization and to select a most suitable filter that provides higher contrast for a range of defects, a large number of phantom images were reconstructed by applying a series of cutoff frequencies, orders, PSF and the noise to signal values for the listed filters. SPECT filters can greatly affect the quality of clinical images by their degree of smoothing. Determining the best filter and the proper degree of smoothing can help to ensure the most accurate diagnosis.

Patients and the reconstructed images were selected randomly and made sure that CT-Angio or angiography of the patients was available for assessment. We acknowledge that reconstruction filter is not the only parameter that affects the image quality and other influencing factors such; scatter, attenuation, partial volume effect, the imaging system calibrations, and choice of collimators are influencing the outcome that is not with the remit of this study. The authors investigated the practical application of the range of filters that were currently employed for cardiac processing at the imaging center, though the imaging system has been regularly maintained to meet manufacturer requirements. Patient preparation and image acquisition protocols were kept unchanged during the period. SPECT/CT images and CT-Angio and angiography performed within 7-days period. At the time of reporting, for both approaches, the nuclear medicine experts were blinded to the results of the CT-Angio and Angiography. The correlation of CAD diagnosis accuracy, using the Wiener filter covered the small heart and those of female patients (28 out of 92 patients). It is noted that false negative reporting dramatically reduced to 7% for moderate to severe CAD cases and to 1% for the mild CAD in comparison to the CT–Agio or Angiography. The outcome has had an impact on follow up treatment and assessment of cardiac patients.

In addition, the results can also help speed image processing time since a proper filter function is often chosen clinically by the tedious and time-consuming process of trial and error. The optimum Wiener filter appears to produce a higher contract for variable size sphere in a uniform background.

The authors would like to underline that the presented results were based on a retrospective study and currently we are collecting patient data based on the prospective study to reconfirm our observations.

## 5 Conclusion

Based on a chest phantom imaging and also reanalyzing and reevaluation of 92 male and female patients (41–77 years old) with suspected CAD, it appears the Wiener filter could produce results with the highest contrast for both cold and hot spheres and for the patient data it produces elevated improvement in diagnostic accuracy and the processed images are consistent with angiography results and correlated well with the CT-Angio and angiography results (*r* = 0.79) and were statistically significant (*P* < .001). Sensitivity of the Wiener filter (0.96) is suggesting high probability that Wiener filter will correctly diagnose CAD patient in comparison to the Butterworth with a sensitivity of 0.69. However, the optimum parameters obtained for the filters have no relation with the resolution of the imaging system but the details of the objects could be improved.

## Acknowledgment

Authors would like to thank Dr Haytham Ramzy at the Radiology – Adan for discussion in relation to angiography and CT- angiography reports and Mrs Aisha Al-Qattan for assistance in the provision of NM data.

## Author contributions

**Conceptualization:** Michael A Masoomi.

**Data curation:** Michael A Masoomi.

**Formal analysis:** Michael A Masoomi, Khaled Kalafallah, Osama Ragab, Jehan Al-Shammari, Sharif Arafat.

**Investigation:** Michael A Masoomi, Iman Al-Shammeri, Hany MA Elrahman.

**Methodology:** Michael A Masoomi, Iman Al-Shammeri.

**Project administration:** Michael A Masoomi, Ebba Ahmed.

**Resources:** Hany MA Elrahman.

**Supervision:** Michael A Masoomi, Sharif Arafat.

**Validation:** Michael A Masoomi, Iman Al-Shammeri, Hany MA Elrahman.

**Visualization:** Michael A Masoomi, Khaled Kalafallah.

**Writing – original draft:** Michael A Masoomi, Iman Al-Shammeri, Khaled Kalafallah.

**Writing – review and editing:** Michael A Masoomi.

## References

[1] Hull DM Peskin CS Rabinowitz AM The derivation and verification of a non-stationary, optimal smoothing filter for nuclear medicine image data. Phys Med Biol 1990;35:1641–62. 2284335 10.1088/0031-9155/35/12/005

[2] Lyra M Ploussi A Filtering in SPECT image reconstruction. Int J Biomed Imaging 2011;2011:1–4. 10.1155/2011/693795 PMC3132528 21760768

[3] King MA Long DT Brill BA SPECT volume quantitation: influence of spatial resolution, source size and shape, and voxel size. Med Phys 1991;18:1016–23. 1961141 10.1118/1.596737

[4] Fakhri GE Buvat I Benali H Relative impact of scatter, collimator response, attenuation, and finite spatial resolution corrections in cardiac SPECT. J Nucl Med 2000;41:1400–8. 10945534

[5] Salihin MN Zakaria A Determination of the optimum filter for qualitative and quantitative 99mTc myocardial SPECT imaging. Iran J Rad Res 2009;6:173–82.

[6] Global Atlas on Cardiovascular Disease Prevention and Control. Eds: WHO; World Heart Federation; World Stroke Organization 2011, ISBN:979 92 4 1564373.

[7] Shaw LJ Iskandrian AE Prognostic value of gated myocardial perfusion SPECT. J Nucl Cardiol 2004;11:171–85. 15052249 10.1016/j.nuclcard.2003.12.004

[8] Berman DS Kang X Slomka PJ Underestimation of extent of ischemia by gated SPECT myocardial perfusion imaging in patients with left main coronary artery disease. J Nucl Cardiol 2007;14:521–8. 17679060 10.1016/j.nuclcard.2007.05.008

[9] Fujimoto S Wagatsuma K Uchida Y Study of the predictors and lesion characteristics of ischemic heart disease patients with false negative results in stress myocardial perfusion single-photon emission tomography. Circ J 2006;70:297–303. 16501296 10.1253/circj.70.297

[10] Lue YH Wacker FJ Cardiovascular Imaging. London, UK: Manson Publishing; 2010.

[11] Sadremomtaz A Taherparvar P The influence of filters on the SPECT image of Carlson phantom. J Biomed Sci Eng 2013;6:291–7.

[12] Hansen CL Digital image processing for clinicians, part II: filtering. Nucl Cardiol 2002;9:429–37. 10.1067/mnc.2002.122898 12161720

[13] Boellaard R Krak NC Hoekstra OS Effects of noise, image resolution, and ROI definition on the accuracy of standard uptake values: a simulation study. J Nucl Med 2004;45:1519–27. 15347719

[14] Salihin MN Zakaria Z Relationship between the optimum cut off frequency for Butterworth filter and lung-heart ratio in 99mTc myocardial SPECT. Iran J Radiat Res 2010;8:17–24.

[15] Smith SW The Scientist and Engineer's Guide to Digital Signal Processing. Santiago, California, USA: California Technical Publishing; 1999.

[16] Gutman F Gardin I Delahaye N Optimisation of the OS-EM algorithm and comparison with FBP for image reconstruction on a dual-head camera: a phantom and a clinical 18F-FDG study. Eur J Nucl Med Mol Imaging 2003;30:1510–9. 14579091 10.1007/s00259-003-1246-6

[17] Knoll P Mirzaei S Mullner A An artificial neural net and error backpropagation to reconstruct single photon emission computerized tomography data. Med Phys 1999;26:244–8. 10076982 10.1118/1.598511

[18] Suzuki S Spatially limited filters for the two – dimensional convolution method of reconstruction, and their application to SPECT. Phys Med Biol 1992;37:37–52.

[19] Miller TR Rollins ES A practical method of image enhancement by interactive digital filtering. J Nucl Med 1985;26:1075–80. 2993553

[20] Taylor D Filter choice for reconstruct tomography. Nucl Med Commun 1994;15:857–9. 7870390 10.1097/00006231-199411000-00001

[21] Gilland DR Tsui BMW McCartnry WH Determination of the optimum filter function for SPECT imaging. J Nucl Med 1988;29:643–50. 3259624

[22] Rajabi H Pant GS Optimum filtration for time-activity curves in nuclear medicine. Nucl Med Commun 2000;21:823–8. 11065155 10.1097/00006231-200009000-00007

[23] Sankaran S Frey EC Gilland KL Optimum compensation method and filter cut-off frequency in myocardial SPECT: a human observer study. J Nucl Med 2002;43:432–8. 11884505

[24] King MA Gilck SJ Penney BC Interactive visual optimization of SPECT pre-reconstruction filtering. J Nucl Med 1987;28:1192–8. 3496435

[25] Trayanova N Computational cardiology: the heart of the matter. ISRN Cardiol 2012;2012:1–5. 10.5402/2012/269680 PMC3505657 23213566

[26] Varga J Bettinardi V Gilardi MC Evaluation of pre- and post-reconstruction count dependent Metz filters for brain PET studies. Med Phys 1997;24:1431–40. 9304571 10.1118/1.598031

[27] Tasdemir B Balci T Demirel B Comparison of myocardial perfusion scintigraphy and computed tomography (CT) angiography based on conventional coronary angiography. Nat Sci 2012;4:976–82.

[28] Di Cesare E Gennarelli A Di Sibio A Assessment of dose exposure and image quality in coronary angiography performed by 640-slice CT: a comparison between adaptive iterative and filtered back-projection algorithm by propensity analysis. Radiol Med 2014;119:642–9. 24553783 10.1007/s11547-014-0382-3

[29] Di Cesare E Gennarelli A Di Sibio A Image quality and radiation dose of single heartbeat 640-slice coronary CT angiography: a comparison between patients with chronic atrial fibrillation and subjects in normal sinus rhythm by propensity analysis. Eur J Radiol 2015;84:631–6. 25617080 10.1016/j.ejrad.2014.11.035

[30] Schuijf JD Bax JJ CT angiography: an alternative to nuclear perfusion imaging? Heart 2008;94:255–7. 18276806 10.1136/hrt.2006.105833

[31] Nissen SE Limitations of computed tomography coronary angiography. J Am Coll Cardiol 2008;52:2145–7. 19095131 10.1016/j.jacc.2008.09.017

[32] Patel MR Detecting obstructive coronary disease with CT angiography and noninvasive fractional flow reserve. JAMA 2012;308:1269–70. 22922590 10.1001/2012.jama.11383

